# Microbiome and Probiotics in Acne Vulgaris—A Narrative Review

**DOI:** 10.3390/life12030422

**Published:** 2022-03-15

**Authors:** Karolina Chilicka, Iwona Dzieńdziora-Urbińska, Renata Szyguła, Binnaz Asanova, Danuta Nowicka

**Affiliations:** 1 Department of Health Sciences, University of Opole, 45-040 Opole, Poland; iwona.dziendziora@uni.opole.pl (I.D.-U.); renata.szygula@uni.opole.pl (R.S.); 2 Medical College Yordanka Filaretova, Medical University of Sofia, 1606 Sofia, Bulgaria; b.asanova@mc.mu-sofia.bg; 3 Department of Dermatology, Venereology and Allergology, Wrocław Medical University, 50-368 Wrocław, Poland; danuta.nowicka@umed.wroc.pl

**Keywords:** acne, microbiome, skin microflora, probiotics, skin care

## Abstract

Acne vulgaris is a chronic disease characterised by the appearance of eruptions such as whiteheads, blackheads, pustules, papules, and cysts. Among factors that cause acne vulgaris are the abnormal keratinisation of the sebaceous canal, bacterial colonisation (*Cutibacterium acnes*), increased sebum production, genotypic factors, and hormonal disorders. Treatment is often long and tedious, and can lead to a reduction in quality of life and social isolation. The intestinal microbiota is greatly important in the formation of acne lesions. It is also responsible for the proper immunity of the organism. Acne is a disease that can be related to the condition of the digestive tract and its microbiome. Research shows that the use of probiotics may reduce skin eruptions. The probiotic supplementation and cosmetics markets are very dynamically developing. The use of internal supplementation and probiotic-containing cosmetics gives hope for the improvement of the skin condition of people with acne.

## 1. Introduction

Acne vulgaris is a disease characterised by skin eruptions such as whiteheads, blackheads, pustules, papules, and cysts. Acne vulgaris can have either noninflammatory or inflammatory lesions, or a mixture of both. Factors that cause this disease can be bacterial colonisation, increased sebum production, and the abnormal keratinisation of the sebaceous canals. Acne vulgaris affects 95% of boys and 83% of girls at the age of 16. In females, acne is more persistent and commonly located on the face, while males experience more severe forms with eruptions located mostly on the chest and back. Acne occurs also in the adult population, not only in teenagers [1].

Standard acne therapy is the most appropriate first-line therapy for noninflammatory comedones or mild inflammatory disease. Topical agents are most frequently used. This group includes keratolytics, alpha-hydroxy acids, benzoyl peroxide, retinoid analogues, azelaic acid, and topical antibiotics. For patients with refractory lesions to topical therapy or those with more severe or extensive disease, oral antibiotics are useful. Tetracyclines, erythromycin, trimethoprim, and trimethoprim/sulfamethoxazole have all been used to inhibit *Cutibacterium acnes*. Tetracycline and erythromycin also have an anti-inflammatory effect in vitro by decreasing neutrophil chemotaxis and chemotactic factors. Patients with severe acne are frequently treated with oral isotretinoin (13-cis-retinoic acid), often with dramatic results. Isotretinoin is an ideal medication based on its mechanisms of action, which include a reduction in sebum production, the inhibition of comedone formation, and the modulation of the inflammatory response; a course of therapy may lead to prolonged remission even in the most severe cases. Unfortunately, isotretinoin is associated with a wide array of adverse effects, and only practitioners who are experienced with its use should prescribe it. It may cause arthralgias, stiffness, tendonitis, serum lipid abnormalities, alopecia, photosensitivity, mucosal and skin dryness, liver function test abnormalities, pancreatitis, depression, anaemia, and leukopenia. Laboratory tests, including serum cholesterol, triglycerides, high-density lipoprotein, liver function tests, and a complete blood count, should be performed before the initiation of isotretinoin therapy and periodically during treatment, generally every 4–6 weeks. Of particular concern is the risk to pregnant women, as isotretinoin is associated with spontaneous abortion and congenital malformations. Its teratogenicity mandates that appropriate screening and counselling be conducted before initiating therapy in women of childbearing age. Urine or serum pregnancy tests should be performed in the week before starting therapy and should monthly throughout treatment. The use of two methods of birth control is recommended; contraception should be practised from 1 month before starting therapy until 1 month after it is discontinued.

As a result of long-term treatment and severe forms of acne, scars can remain on patient skin and reduce quality of their life. Symptoms caused by acne contribute to the development of depression, especially in adolescents. Post inflammatory hyperpigmentation can cause fears that lead to deterioration of quality of life. It is very important to contact a dermatologist who could professionally assess the causes of acne and select the appropriate treatment that prevents complications such as discoloration or acne scars [2].

Androgen hormones and testosterone control sebum production. Patients with severe acne, both men and women, have increased levels of dehydroepiandrosterone sulphate, sex hormone-binding globulin, and androstenedione. Excessive sebum production plays a key role in the pathophysiology of acne and generates the inflammatory process [3].

Researchers claim that there is a relationship between acne and the consumption of milk or other dairy products. There is the hypothesis that consuming highly glycaemic food and milk can increase the level of insulin. This can lead to inflammation, androgenic stimulation, and the formation of blackheads and whiteheads [4].

## 2. Skin Microbiome

The term microbiome (microbiota) describes the entirety of microorganisms present in a given habitat. The skin is the most external organ of our body and is inhabited by bacteria, viruses, fungi, and mites. Most of the microorganisms that inhabit the skin are harmless to the skin and live in symbiosis with skin cells [5,6,7,8].

The main types of dermal colonisation are the following bacteria: *Actinobacteria* (*Micrococcus* spp.), Firmicutes (nonhaemolytic aerobes and anaerobes), *Staphylococci* (*Staphylococcus* spp.), *Clostridium* spp., ahaemolytic *Streptococci* (*Streptococcus* spp.), *Enterococci* (*Enterococcus* spp., *Sphingobacterium* spp., *Chryseobacterium* spp.), *Proteobacteria* (*Janthinobacterium* spp., *Serratia* spp., *Halomonas* spp., *Delftia* spp., *Comamonas* spp.) [9,10]. Bacteria inhabiting the surface of the skin include *S. epidermidis*, *S. saprophyticus*, *S. hominis*, *S. warneri*, *S. haemolyticus*, and *S.capitis*, bacteria of the genus *Cutibacterium* (*C. acnes*, *C. jeikeium*) and bacteria of the genus *Micrococcus* (*M. luteus*, *M. varians*, *M. lylae*, *M. sedentarius*, *M. roseus*, *M. kristinae*, and *M. nishinomiyaensis*). The skin can also be inhabited by pathogenic bacteria such as group A *Streptococci* (*S. pyogenes*) and Gram-negative bacilli (*P. aeruginosa*) [11,12].

On the surface of human skin, there are also fungi of genera *Malassezia* (*M. furfur*, *M. sympodialis*, *M. globosa*, *M. restricta*, *M. slooffiae*, *M. yamatoensis*, *M. obtusa*, *M. dermatis*, and *M. japonica*), *Penicillium* (*P. chrysogenum*, *P. lanosum*), *Aspergillus* (*A. candidus*, *A. terreus*, *A. versicolor*), *Candida* (*C. tropicalis*, *C. parapsilosis*, *C.orthopsilosis*), *Chaetomium*, *Chrysosporium*, *Cladosporium*, *Mucor*, *Debaryomyces*, *Cryptococcus* (*C. flavus*, *C. dimmennae*, *C.diffluent*), *Trichophyton* and *Rhodotorula*. Pathogenic fungi that are capable of causing skin diseases include the following dermatophytes (*Microsporum*, *Trichophyton*, *Epidermophyton*) [9,13,14,15,16].

As mentioned above, the skin microbiome also includes mites. *Demodex* spp. are characteristic of sebaceous glands and hair follicles, with the most numerous representatives being *D. folliculorum* (hair follicles), *D. brevis* (sebaceous glands and Meibomian glands). *Demodex* mites are carriers of a proinflammatory Gram-negative bacterium *Bacillus oleronius*, which is considered to cause inflammatory responses [17].

The skin virome has been investigated, but it constitutes a large part of the skin microbiome, which is due to the difficulties associated with viral sequencing [13]. The viral skin flora is complex and heterogeneous with multiple polyomaviruses (*Polyomaviridae*), circoviruses (*Circoviridea)*, and papillomaviruses (*Papillomaviridae*) detected on healthy skin [18,19,20]. The gut microbiome also contains a small proportion of both DNA viruses (*Anelloviridae*, *Geminiviridae*, *Herpesviridae*, *Nanoviridae*, *Papillomaviridae*, *Parvoviridae*, *Polyomaviridae*, *Adenoviridae* and *Circoviridaeas*) andRNA viruses (*Caliciviridae*, *Picornaviridae*, *Reoviridae*) [21]. The human virome also comprises phagesthat appear in the microbiome very early in life. They play an important role in the regulation of the bacterial microbiota. The most impotent phage genes are *Caudovirales* and *Microviridae* [21,22].

Little is known about *Archaea*. Recent studies using quantitative PCR of archaeal gene sequencing found *Archeae* accounting for up to 4.2% of the total skin microbiome. Most of the *Archeae* are *Thaumarchaeota* [23].

Bacterial composition depends on the use of cosmetics, environmental factors, profession, exposure to UV radiation, antibiotics, and humidity [24,25,26]. The microbiome landscape of the skin changes with age; in newborns, the skin microbiome largely depends on the type of delivery, while in children, *Firmicutes* becomes dominant. Gender also contributes to the composition of the skin microbiome. The surfaces of the hands and forearms of women are colonised by more diverse microorganisms, while men have more *Malassezia* than women do. These elements are influenced by make-up, among other things [24,27]. Environmental and endocrine factors specifically influence the virulence of *C. acnes* [28,29]. In recent times, the skin microbiome has often been analysed in relation to the etiopathogenesis of dermatological diseases. Atopic dermatitis, psoriasis, rosacea, and hidradenitis suppurativa play the best-known roles in the microbiome.

Recently, the relationship between clinical manifestations of atopic dermatitis and the skin microbiome of atopic patients has been analysed. Research shows that pathological microflora dominates in patients with exacerbations of the disease and severe lesions. Conversely, the commensal microflora appeared again on the skin with clinical improvement. Furthermore, a dysfunction of the skin barrier function observed in patients with atopic dermatitis facilitates the colonisation by *S. aureus*, which translates into a numerical increase in its colonies on the skin of affected patients. *S. aureus* can modulate inflammatory responses via mediators; and thus, control inflammation. The surface of the skin and the intestines participates in the development of allergy or asthma due to the immunological pathways of these organs. Changes in the skin microflora with age promote the maturation of the immune system by providing epitopes for immunity-boosting training. Other factors responsible for changes in the skin microbiome include topical medications (corticosteroids, immunomodulators) and topical preparations (moisturisers, emollients) recommended for primary and secondary prophylaxis. Overall, stimulating the growth of symbiotic bacteria can restore the balance of the microbiome, thereby reducing the symptoms of inflammation in atopic patients. The presence of Gram-positive bacteria on the skin (*Streptococcus*, Lactococcus, and *Streptomyces*) restrains the spread of other pathogenic bacteria. For this reason, some emollients containingsymbiotic bacteria improve skin condition, acting similarly to probiotics.

A link between the skin microbiome and psoriasis has also been observed. Current research highlights that patients with psoriasis have a different skin microbiome than that of healthy people. Studies have shown that some microorganisms can alter the course of the disease, for example, bacteria including *S. aureus* and *S. pyogenes*, viruses (*Papillomaviridae* and endogenous *Retroviridae*), and fungi (*Malassezia* spp. and *Candida albicans*). Interestingly, high concentrations of *Malassezia* spp. detected in the lesion of patients with psoriasis resulted in an increased risk of irritation when the skin was treated with topical calcipotriol. Furthermore, antifungal agents improve the condition of nail plates in psoriasis, which may be caused by the elimination of fungi and their ability to worsen skin lesions. Phototherapy, with its ability to improve oxidative stress parameters, could also change the microflora of the skin and improve its condition. The association among psoriasis, changes in skin microbiota, and systemic oxidative stress parameters after narrowband UVB therapy is an interesting topic of further research.

The exact pathophysiology of rosacea is still poorly understood, but current theories focus on the role of the cutaneous microbiome, specifically *D. folliculorum* and a few commensal bacteria, in the propagation of an inflammatory response. However, the theory of microbial induction extends beyond the skin to include the gastrointestinal microbiome and complications therein. Additional gastrointestinal pathologies have been implicated, including infection by *H.pylori* and irritable bowel syndrome. The treatment of rosacea with topical antibiotics and antiparasitic agents has long reigned supreme, followed by oral agents of the tetracycline class. Future studies should further investigate the role of the gastrointestinal microbiome in the pathogenesis of rosacea, as intraluminal agents such as rifaxim in have beneficial effects.

In hidradenitis suppurativa, also called acne inversa, dysregulated microbiome is mentioned among other causative factors [30]. Research reports differences between lesional and nonlesional skin in people with and without hidradenitis suppurativa in terms of the composition of the skin microbiome. In patients with hidradenitis suppurativa, the microbiome of skin lesions included *Cutibacterium* species, *Porphyromonas* and *Peptoniphilus* species, while in the microbiome of nonlesional skin, *Acinetobacter* and *Moraxella* species dominated. No *C. acnes* was found in patients with hidradenitis suppurativa [31]. Evidence of differences in the composition of the gastrointestinal microbiome is limited but suggests that the gut microbiome of patients with hidradenitis suppurativa may be altered when compared to that of healthy people [32].

## 3. Skin Microbiota in Acne

*Cutibacterium acnes* is considered to be the most likely acne pathogen. Its classification and terminology (former names *Propionibacterium* and *Corynebacterium*) were updated by the Microbiology Society [33,34]. *C. acnes* is a bacterium found in areas rich in sebum, such as the scalp, face, chest, and back. However, most of this bacterium is found on the scalp and face, then on the upper limbs, and the least on the lower limbs. *C. acnes* is observed with age, it rarely occurs on the skin of children, increases in adolescence and adulthood, and then decreases after the age of 50 years [12,35]. Although the role of *C. acnes* in the pathophysiology of acne is not fully understood, its main role is that it helps in keeping the skin pH low by releasing free fatty acids and inhibiting the multiplication of *S. aureus* and *Streptococcus*. The population *of C. acnes* strains is diverse depending on the characteristics of the cohorts of the examined patients. Strains can be classified into ribotypes (RT) on the basis of *C. acnes* 16S rDNA sequences [36]. Fitz-Gibbon et al. showed a strong association between *C. acnes* strains of RT4 and RT5. Strains of RT6 were linked with healthy skin. They also concluded that RT7, RT8, RT9 and RT10, and interactions that may take place among the different strains, may contribute to the development of the disease [37]. *C. acnes*, in comparison with healthy strains, contains additional virulence genes [38]. In contrast, Johnson et al. found that acne-related strains generate more porphyrin, which can cause inflammation in keratinocytes [39]. Association with acne causes inflammation in keratinocytes, peripheral blood mononuclear cells and sebocytes, while strains in healthy patients do not [40,41], and hyperkeratinisation of hair sebaceous units [42,43]. *C. acnes* stimulates the innate and specific response. It expresses pattern recognition receptors (PRRs) and receptors from the toll-like receptor (TLR) family, of which TLR-2 and TLR-4 play a key role. The TLR-2 receptor activates the NF-B pathway and produces cytokines such as Il-1, Il-6, Il-8, IL-10, Il-112, and TNF. Cytokines IL-1 and IL-6 stimulate the proliferation of keratinocytes, Il-1 is a proinflammatory cytokine and increases the production of sebum and reduces the content of linoleic acid. Proinflammatory factors activate transcription factor Ap-1, which induces the gene for matrix metalloproteinase. This process is followed by the synthesis of metalloproteins involved in the destruction of connective tissue [44]. *C. acnes*, due to the presence of lipase, hydrolyses di- and triglycerides that are part of sebum into free fatty acids, have an irritating and proinflammatory effect, and intensify follicular keratosis. Additionally, *C. acnes* produces protease hyaluronidase and neuraminidases, which also have a proinflammatory effect. In the pathogenesis of acne, insulin growth factor 1(IGF-1) stimulating the production of sebum also plays a role. It increases skin eruptions in women, while in men, it causes seborrhoea [45]. *C. acnes* and a hyperglycaemic diet stimulate the proliferation of keratinocytes and the formation of comedones [46].

Human skin is colonised by many microbes, and although *C. acnes* is most associated with acne eradication, other microbes also influence the development of this disease. On the basis of scientific studies, *C. granulosum* is more virulent than *C. acnes*, and is found in comedones and pustules in acne patients [47]. *Malassezia* is also believed to be an acne-causing organism [48]. Many studies showed that *Malassezia* also hydrolyses triglycerides to free fatty acids, which cause hyperkeratinisation of the hair follicle ducts and the formation of comedones [49].

## 4. Influence of Intestinal Microbiota on Acne Lesions

The intestinal microbiota is greatly important in the formation of acne lesions. It is also responsible for the proper immunity of the organism and defence by microorganisms, thus determining tolerance to substances supplied to the human body with food, leading to an immune response [50]. Acne and the condition of the digestive tract are both associated with the quality of microbiome inhabiting intestines. In addition, both skin and the intestines are very densely vascularised and innervated. They perform neuroendocrine and immune functions, among others. An increasing number of studies indicate that the health of the intestines is related to the health of the skin [51,52]. Research on the microbiota is still incomplete, as what influence it has on the condition of the skin has not been fully investigated. The Western diet disturbs the balance between beneficial and pathogenic microorganisms, which contributes to inflammation, including inflammatory skin diseases. Stress also disturbs eubiosis, and bacteria *Lactobacillus* and *Bifidobacterium* are particularly sensitive to its effects. In stressful situations, microorganisms can produce neurotransmitters that are inflammatory for the body. The microbiota also modifies the production of short-chain fatty acids (SCFAs), which fulfil many functions, e.g., nourishing intestinal cells and modulating brain activity. One such acid is propionic acid, which is toxic to *S. aureus*. Hence, SCFAs may also affect the skin’s resistance to cutaneous *Staphylococcus* and *C. Acnes* [53,54]. However, the gut microbiota is still a poorly understood topic, and only a few scientists have studied the gut flora of acne patients, as shown in Table 1.

Although knowledge on the human microbiome has significantly expanded in recent years, the microbiological environment in humans is complex and unique. Over the past several decades, researchers have searched for the relationship between the microbiota and *C. acnes*. Recent studies have shown that *C. acnes* strains dominate in acne vulgaris, but all limitations and methods of skin sampling indicate the need for more extensive research in this area.

## 5. Externally Used Probiotics in Dermatology and Cosmetology

Probiotics are defined as “live microorganisms that, when administered in adequate amounts, confer a health benefit on the host” [59]. They do not show carcinogenic effects, but instead show a protective effect and are safe to use. The best-known microorganisms with probiotic effects are *Lactobacillus* and *Bifidobacterium*. They belong to anaerobic bacteria, are normal micro white flora, and are Gram-positive. In order for probiotic microorganisms to be safe for human health, they must be nonpathogenic and nontoxic [59,60,61]. As early as 1900, Louis Pasteur defined the microorganisms responsible for fermentation and stated that “lactobacilli might counteract the putrefactive effects of gastrointestinal metabolism that contributed to illness and ageing” [62].

Probiotics may be used in various product categories such as drugs, cosmetics, dietary supplements, and food and food additives. Their use in oral preparations is well-regulated, while their use in cosmetics still requires to be regulated. There is no legal regulation for topical probiotics [63,64]. According to the FDA, a cosmetic is “a product (excluding pure soap) intended to be applied to the human body for cleansing, beautifying, promoting attractiveness, or altering the appearance” [65], yet probiotics are living microorganisms that are ingested [59]. The International Cooperation on Cosmetics Regulation (ICCR) developed categories of microbiome-related products that included probiotics and postbiotics. Postbiotics were defined as “preparations of inanimate microorganisms and/or their components that confer a health benefit on the host”. They can also be used on a host surface, including the mucosa and skin [66,67]. The action of probiotics is based on binding them to the epidermal surface, inhibiting pathogens, producing antimicrobial substances, and increasing immunomodulatory properties. Immunomodulatory properties transformthe probiotic cosmetic into a preparation that can be used in the case of dermatological skin diseases. Probiotics began to be used as an ingredient in intimate hygiene products, hair shampoos, creams, and toothpaste. The most common strains that probiotic cosmetics contain include *Bacillus sabtilis*, *Lactobacillus acidophilus*, *Lactobacillus casei*, *Lactoccocuslactis*, and *Lactobacillus plantarum*. These probiotics have a deep moisturising effect, stimulating the production of lipids, and repair processes in the epidermis. The benefits of their use are as follows: the number of unfavourable pathogens is eliminated or reduced, the production of toxic metabolites is reduced, the production of antibodies is increased, the homeostasis of the immune system is restored, and the synthesis of the cytokines is regulated [68,69,70,71].

When it comes to the use of probiotics in cosmetology and dermatology, these are mainly products for washing and caring for skin with atopic dermatitis, acne-prone skin, skin with eczema, psoriasis, and after invasive treatments in the field of cosmetology or medicine, e.g., exfoliation with acids. Acne is a disease that manifests itself in the appearance of blotchy skin eruptions. Astudy by Di Marzio et al. showed that the external application of *Streptococcus thermophilus* to the skin for a period of 7 days increased the production of ceramides [72]. Other researchers found that these studies were helpful in the treatment of acne, as ceramides such as phytosphingosine provide antimicrobial and anti-inflammatory activity against *Cutibacteriumacnes* [73]. Kang et al. tested the action of lotion with *E. faecalis* with mild-to-moderate acne. The study exhibited diminution of papules and pustules among patients, and showed that it could be an alternative treatment to typical antibiotics. Topically applied probiotics can act as a protective shield, preventing colonisation by other pathogens [74,75].

For several years in cosmetology, probiotics have been added to everyday care products. Their main role is to protect against harmful pathogens and reduce inflammation [76]. Fragments of cell walls and inanimate bacteria are contained in such cosmetic products as serums, ointments, creams, body balms, body gels, and shampoos. Products with antiwrinkle, antiageing, and moisturising properties are used thanks to lactic acid. The use of such products promotes the regeneration of damaged skin, e.g., as a result of excessive sunbathing. Probiotics also protect the skin of the hands and the nail plate, and have an anti-itching effect. Research shows that the use of probiotics also has a favourable effect on the regulation of pH thanks to the use of facial cleansing fluids, peels, and toning preparations [77,78]. The use of preparations influences the development of normal microflora, thanks to which a protective barrier is created. The epidermis maintains adequate moisture and strengthens the skin’s resistance to all negative external factors. The use of bacteria such as *Streptococcus salivarium* spp. and *S. thermophilus* S244 in cosmetics leads to the production of enzymes that moisturise the skin, thus reducing skin dryness and slowing down ageing processes [79].

## 6. Oral Probiotics for Acne Vulgaris

So far, little research exists on the effect of probiotics on acne-prone skin. However, there are studies that show the positive impact of their use. Jung et al. investigated 45 women taking the minocycline antibiotic. They examined whether probiotic supplementation would reduce side effects and whether there would be a synergistic effect when using these two products. The participants were divided into 3 groups: the first group received a probiotic, the second group received an antibiotic, and the third group received an antibiotic and a probiotic. After completing the research, the scientists deduced that probiotics could be considered to be a therapeutic or auxiliary option in the treatment of acne. There was a synergistic anti-inflammatory effect, while probiotics minimised the appearance of side effects resulting from the use of antibiotic therapy [80]. In 2010, the ability of konjac glucomannan hydrolysates and probiotics (*L. casei*, *L. plantarum*, *L. gasseri*, *L. lactis*) to inhibit *C. acnes* was investigated. Studies showed that all tested strains and konjac glucomannan hydrolysates significantly inhibited the growth of bacteria. The researchers recommended further research in terms of the therapeutic or prophylactic use of the above-mentioned synbiotics [81].

Kang et al. conducted a study in which the role of *E. faecalis* SL-5 on *C. acnes* was examined. Aspecial concentrated powder of CBT SL-5 was prepared that used cell-free culture supernatant from *E. faecalis* SL-5. The study concluded that this bacteriocin was able to reduce inflammation. The researchers suggested that *E. faecalis* can be an alternative option in future acne therapy [74]. In another study that ran over a two-month period, 15 females were recruited. Patients used a base cream on the left side of their face, and a base cream containing heat-killed *L. plantarum*-GMNL6 (1 × 10^9^ cells/g of cream)on the right side. The face quality was checked, and moisturising and skin colour tests were conducted. The E (erythema) and M (melanin)indices were determined. The hydration level of the skin was checked before and after the study. DNA was extracted from the head. After completion of the research, i*L. plantarum*-GMNL6 enhanced collagen synthesis and the gene expression of serine palmitoyltransferase small subunit A. It reduced melanin synthesis, a biofilm of *S. aureus*, and the proliferation of *C. acnes*. Skin moisture, spots, and porphyrins were improved [82].

Espinoza-Monje et al. were the first who characterised the antimicrobial and immunomodulatory features of *Weissellaviridescens* UCO-SMC3. The scientists isolated lactic acid bacteria from garden-culture (Helix aspersa Müller) slime and selected *W. viridescens* UCO-SMC3. Their study showed that the UCO-SMC3 strain is resistant to unfavourable gastrointestinal conditions. Oral and topical administration of the strains was used, which lead to a favourable modulation of the inflammatory response. The cream applied to volunteers with acne vulgaris reduced skin eruptions [83]. Korean researchers used the synergyof probiotic lactic acid bacteria (LAB) with curcuma longa extract (CLE) as a symbiotic against *C. acnes*. They concluded that the combination of probiotic LAB and CLE produced cooperation antibacterial effects against *C. acnes*, which gives hope for the use of these preparations in the medical and cosmetic industries as preparations to treat or soothe acne vulgaris [84].

## 7. Conclusions

Acne is a skin disease with an inflammatory background. Using antibiotics or isotretinoin has a negative effect on the intestinal flora. Patients with severe acne are frequently treated with oral isotretinoin (13-cis-retinoic acid), often with dramatic results. Isotretinoin is an ideal medication based on its mechanisms of action, which include a reduction in sebum production, the inhibition of comedo formation, and modulation of the inflammatory response; a course of therapy may lead to prolonged remission even in the most severe cases. Unfortunately, isotretinoin is associated with a wide array of adverse effects, and only practitioners who are experienced with its use should prescribe it. It may cause arthralgias, stiffness, tendonitis, serum lipid abnormalities, alopecia, photosensitivity, mucosal and skin dryness, liver function test abnormalities, pancreatitis, depression, anaemia, and leukopenia. Scientists are constantly looking for new solutions that would bring good results when it comes to the treatment of acne. Research shows that the use of probiotics may reduce skin eruptions. The intestinal microbiota is greatly important in the formation of acne lesions. It is also responsible for the proper immunity of the organism. Acne is a disease that can be linked to the condition of the digestive tract and its microbiome. Research shows that the use of probiotics may reduce skin eruptions. The probiotic supplementation and cosmetics markets are dynamically developing. The use of internal supplementation and cosmetics with probiotics give hope for the improvement of the skin condition of people suffering from acne.

## Figures and Tables

**Table 1:** Summary of available studies on gut flora in acne patients.

Author and Study Year	Research Conclusions
Loveman et al., 1955 [55]	People with acne show a significantly different intestinal flora compared to the control group.
Volkova et al., 2001 [56]	Acne patients showed less variability in the gut microflora and a higher ratio of *Bacierioidetes* to Firmicutes being an entero type of the Western diet.
Deng et al., 2018 [57]	Reduction in the number of *Bifidobacterium*, *Lactobacillus*, *Coprobacillus*, *Butyricicoccus*, and *Allobusulum* in acne patients compared to the control group.
Yan et al., 2018 [58]	*Bacterioidetes* spp. that escalated under stressful conditions were isolated in acne patients.

## Data Availability

Not applicable.
